# Supplementary material for: Transcriptomic Insights into Mechanisms of Early Seed Maturation in the Garden Pea (Pisum sativum L.)
Source: Cells. 2020 Mar 23;9(3):779. doi: 10.3390/cells9030779 (PMC7140803; doi:10.3390/cells9030779)

Sprint−2(20) vs Zhewa

Global Summary

%DE = 0.79

genes with fdr < 0.2 = 483 (249 + / 234 −)

genes with fdr < 0.1 = 426 (220 + / 206 −)

genes with fdr < 0.05 = 354 (173 + / 181 −)

genes with fdr < 0.01 = 254 (109 + / 145 −)

genes in genesets = 0

<FC> = 0

<t−score> = 1242.99

<p−value> = 0

<fdr> = 0.21

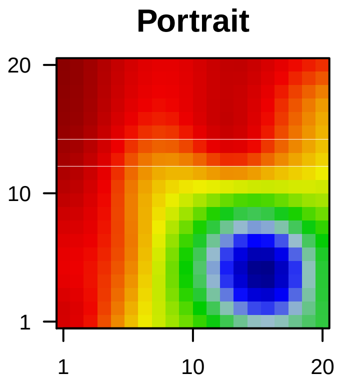

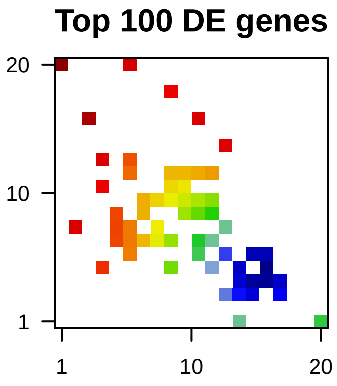

Global Genelist

Rank	ID	log(FC)	p−value	fdr	Metagene	Description
1	TRINITY_DN2	1.69	0e+00	0.0000002	10 x 1	
2	TRINITY_DN2	−0.07	8e−10	0.0000004	13 x 3	
3	comp44710_c	−0.05	7e−09	0.0000004	11 x 9	
4	TRINITY_DN1	0	7e−09	0.000002	9 x 11	
5	TRINITY_DN1	−1.02	4e−08	0.000002	16 x 5	
6	TRINITY_DN5	0	7e−08	0.000002	8 x 10	
7	TRINITY_DN2	0	9e−08	0.000002	9 x 11	
8	TRINITY_DN2	0	9e−08	0.000005	9 x 10	
9	TRINITY_DN8	0.02	1e−07	0.000005	8 x 7	
10	TRINITY_DN1	−0.23	2e−07	0.000005	9 x 7	
11	comp39525_c	0.25	2e−07	0.000016	6 x 8	
12	TRINITY_DN2	−0.01	3e−07	0.000016	11 x 6	
13	TRINITY_DN1	−0.03	5e−07	0.000016	14 x 3	
14	TRINITY_DN4	0.65	6e−07	0.000027	14 x 1	
15	TRINITY_DN2	−0.03	9e−07	0.000027	9 x 12	
16	TRINITY_DN5	0	1e−06	0.000027	9 x 11	
17	TRINITY_DN1	0	1e−06	0.000027	6 x 9	
18	TRINITY_DN2	0	2e−06	0.000027	9 x 10	
19	TRINITY_DN1	−1	2e−06	0.000060	16 x 5	
20	TRINITY_DN1	0	2e−06	0.000060	9 x 11	

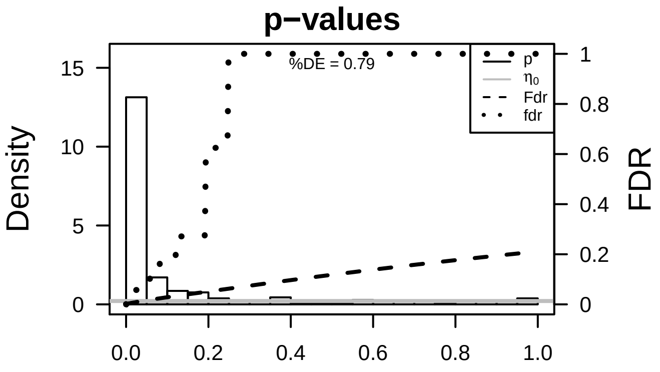

Supplement: Supplementary file 1 [file cells-09-00779-s001.zip › Supplementary materials/FileS3.zip!/FileS2/Hormones - Results/Summary Sheets - Differences/Reports/Sprint.2.20..vs.Zhewan.1.10..pdf]
